# Meta-operators: all optical and wireless image processing via metasurfaces

**DOI:** 10.1038/s41377-026-02318-1

**Published:** 2026-06-02

**Authors:** Lei Xu, Mohsen Rahmani

**Affiliations:** Advanced Optics & Photonics Laboratory, Department of Engineering, School of Science & Technology, Nottingham Trent University, Nottingham, NG11 8NS UK

**Keywords:** Metamaterials, Imaging and sensing

## Abstract

Arrays of resonant nanoparticles, so-called metasurfaces, have been developed and demonstrated as the first generation of meta-operators. Unlike today’s electronic systems, the demonstrated compact, scalable platform enables ultrafast, energy-efficient all-optical image processing, extending to holographic wavefront shaping with a single-layer metasurface. These results open new opportunities for advanced optical computational microscopy and intelligent sensing.

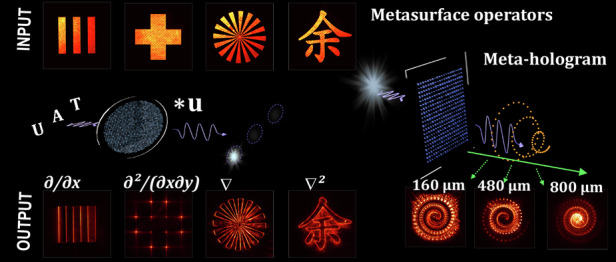

Optical systems, benefiting from the unmatched speed of light, are rapidly advancing as an alternative to conventional electronic systems. In particular, the capability to parallel-process spatially encoded information across multiple lightwave channels has attracted technology entrepreneurs for next-generation image processing and transformation technologies. Unlike electronic processors, which process pixel data sequentially, optical systems can operate on spatially encoded information in a fully passive manner. This enables ultrafast, energy-efficient image processing^1–5^. However, traditional optical processing still relies on bulky optical components, such as lenses, mirrors, and spatial light modulators, which hinder the scalability and integration of miniaturised image processing systems.

In recent years, an array of nanoscale resonators, so-called metasurfaces, has emerged as a powerful tool for manipulating light at the subwavelength scale to achieve both near- and far-field wavefront control for a wide range of applications^6–8^. This has sparked a growing interest in using metasurface for compact optical information processing^9–14^.

However, to achieve full amplitude and phase control for image processing, today’s metasurface-based approaches either require bulky optical setups or multi-metasurface configurations. As a result, regular approaches for implementing metasurfaces are not practical for image processing applications.

In a major demonstration in *Light: Science & Applications*^15^, Yu, Caglayan and colleagues introduced a versatile approach for realising metasurface-based optical image processing. They have demonstrated a compact, scalable platform for all-optical image processing using a single metasurface layer. By leveraging double-phase encoding^16^ and polarisation multiplexing, they have introduced an arbitrary all-optical image transformation, so-called meta-operators. A complex optical field $u\left(x,y\right)$ with amplitude $A(x,y)$ and phase $\theta (x,y)$ is represented as the interference of two phase-only components ${u}_{1}$ and ${u}_{1}$ with equal amplitude expressed as $u\left(x,y\right)=A\left(x,y\right){e}^{i\theta \left(x,y\right)}=B{e}^{i\left(\theta \left(x,y\right)+\psi (x,y)\right)}+B{e}^{i\left(\theta \left(x,y\right)-\psi (x,y)\right)}$, with $B={A}_{\max }/2$ is a constant, and $\psi \left(x,y\right)={\cos }^{-1}\left[A(x,y)/{A}_{\max }\right]$. This allows the realisation of complex modulation using phase-only optical modulation. By implementing a metasurface, the two-phase components can be encoded in two orthogonal polarisation channels within a single layer^17,18^. Subsequently, by employing a polarisation analyser to recombine the channels at the output, the required complex amplitude distribution corresponding to a given optical operator can be reconstructed. By applying this innovative approach, this work demonstrates simultaneous control of amplitude and phase within one single-layer, 100 times thinner than human hair.

The metasurface exploited in this work was composed of 600-nm-thick TiO_2_ nanoparticles, designed and fabricated over an area of 450 μm × 450 μm. The particles are resonant and operational at 532 nm in the visible spectral range. As illustrated in Fig. 1, via these metasurfaces, Yu et al experimentally demonstrated several key image processing functions, including object edge detection and object recognition functions through the first-order spatial differentiation and cross-correlation operations, as well as more complex operations which are essential for higher-order spatial filtering in imaging processing, such as vertex detection and Laplacian differentiation^15^. Importantly, here all these operations are realised directly in the optical domain without any digital post-processing, thereby avoiding any latency and power consumption associated with electronic processing. With computation at the speed of light, this approach is particularly promising for applications that require instantaneous decision-making, such as autonomous vision systems, optical security verification, and real-time object tracking. It can also pave the pathway towards hybrid optical-digital architectures for high-speed image processing.

**Fig. 1:**
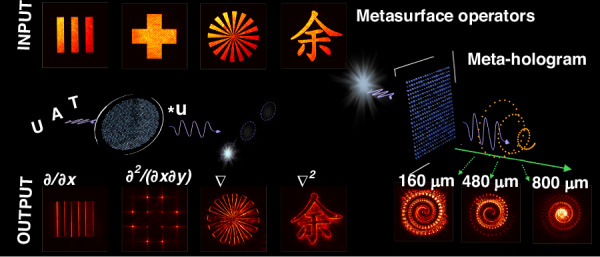
Illustration of the metasurface operators for all-optical imaging processing and complex holography. Adapted from Ref. ^15^

Besides imaging processing, the framework can be extended for complex holography and generate subwavelength-scale volumetric holographic reconstructions with high fidelity.

These results suggest the potential of metasurface operators not only for optical computing but also for advanced wavefront engineering in imaging and displaying technologies. With growing interest in photonic information processing and optical neural network, such compact metasurface operators are playing an increasingly important role for future image and information processing. Looking forward, integration with photonic chips could allow fully integrated optical computing systems^19^. In addition, combining this successful approach with nonlinear and tuneable metasurfaces may further introduce reconfigurable and programmable functionalities for advanced imaging systems.
